# Valorization of Polysaccharides From *Benincasa hispida*: Physicochemical, Moisturizing, and Antioxidant Skincare Properties

**DOI:** 10.3389/fphar.2022.912382

**Published:** 2022-06-17

**Authors:** Qian Wang, Xiaoyan Yang, Changwei Zhu, Guodong Liu, Weili Han, Yujun Sun, Lisheng Qian

**Affiliations:** ^1^ College of Life and Health Sciences, Anhui Science and Technology University, Anhui, China; ^2^ College of Agriculture, Anhui Science and Technology University, Anhui, China

**Keywords:** Benincasa hispida, polysaccharide, skincare, moisturizing, anti-aging, antioxidation

## Abstract

*Benincasa hispida* Cogn. (*B. hispida*) is a popular vegetable in China, and studies have been reported on *B. hispida* polysaccharides (BPS) preparation. However, few studies have been reported on its physicochemical and skincare properties. In this study, we analyzed the physicochemical properties of BPS, free radical scavenging capability, moisturizing and antioxidant activities *in vitro* and *in vivo*, respectively. Our results show that BPS was an inhomogeneous acidic polysaccharide that could scavenge a variety of free radicals. Also, BPS had a good moisturizing and antioxidant capability both *in vitro* and *in vivo*. Specifically, BPS could alter some key antioxidant enzyme activities and pro-inflammatory factor levels via activating the NRF2/HO-1 pathway, thereby preventing H_2_O_2_-induced reactive oxygen species (ROS) production and apoptosis of HDF-1 cells. Our results suggest that BPS exhibited favorable moisturizing and anti-aging properties and might be an attractive candidate for the development of anti-aging skincare products.

## 1 Introduction

*Benincasa hispida* Cogn. (Family: Cucurbitaceae Juss.), native to Asia and also known as *kundur fruit*, *wax gourd*, *tallow gourd*, and *chalkumra*, is considered one of the famous crops of the cucurbit family and has various therapeutic uses in traditional medicine (Islam et al., 2021). Its fruit is known for its nutritional and medicinal properties (Purohit et al., 2019), containing various essential nutrients, including natural polysaccharides, dietary fiber, vitamins, amino acids, organic acids, and mineral elements (Andrias et al., 2019; Purohit et al., 2019). Additionally, some bioactive ingredients such as flavonoids, saponins, and tartaric acid are also contained therein (Jiang et al., 2016). Generally, the mature fruit of *B. hispida* is mainly eaten as an inexpensive and green vegetable. However, this dramatically limits the development of its potential functional features. Recent developments in *B. hispida* have heightened the need for its added value and potential economic benefits. Isolation of specific high-value components, such as water-soluble polysaccharides, may lead to better utilization of this resource.

Natural plant polysaccharides are considered superior functional ingredients and exhibit a variety of excellent bioactivities (Chen et al., 2018; Zhu et al., 2021b), and skincare is one of them. For example, *Marva* nut polysaccharide has superior moisturizing performance, and the developed skin moisturizing gel containing *Marva* nut polysaccharide shows improved performance over benchmark tamarind and algae polysaccharide gels (Kanlayavattanakul et al., 2017). Likewise, another study indicated that tea polysaccharides could effectively scavenge various free radicals (Zhu et al., 2021b). Additionally, polysaccharides from *Laminaria japonica* exhibit excellent anti-melanogenesis capability (Chen et al., 2019). However, the use of this polysaccharide in their bioactivity has, to date, been rarely reported, especially in current skincare applications, and only a few pieces of the literature indicated the structural information of several BPSs (Mazumder et al., 2004). To better utilize this polysaccharide, identifying its physicochemical composition and exploring its skincare activity will help to optimize the management of sub-healthy skin populations and the development of related functional polysaccharide products.

With the improvement of current people’s living standards, people pay more and more attention to skin protection. Skin is the largest tissue in the human body and is vital to human health. It is not only the body’s first barrier of defense against harmful external invasions but also plays an important role as a dynamic tissue in metabolism and the interaction between internal and external cells (Solano, 2020). In addition, the skin has a highly specialized immune niche, and resident immune and non-immune cells in the skin contribute to skin physiology and are essential for maintaining tissue homeostasis, defense, and repair (Nguyen and Soulika, 2019). Unfortunately, with the change in the external environment and the fast-paced lifestyle, a series of skin-related issues have appeared, resulting in the long-term sub-health state of the skin (Taylor et al., 1997). The sub-health state further triggers the damage of the skin’s physical barrier components, resulting in elevated skin inflammation and reduced secretion of antimicrobial peptides (modulating host immunity) (Yuki et al., 2016; Lezi et al., 2018; Nguyen and Soulika, 2019). The raw materials of most skincare products are mainly artificially synthesized and have specific side effects, such as allergy and mutagenicity (Adomaite et al., 2020; Nepalia et al., 2021). Therefore, natural skincare ingredients that are non-toxic and non-polluting are increasingly favored by consumers. Many studies show that plant polysaccharides have great hygroscopic and moisturizing, anti-oxidative, anti-aging, anti-inflammatory, antibacterial, and other bioactivities, and they also have a potential affinity with human skin (Pinzon et al., 2018). These bioactivities provide a broad space for its development and application in the field of officinal skincare products and also prompted us further to investigate the skincare activities and mechanisms of BPS.

In this work, we explored the physicochemical properties of BPS and its various skincare activities, which provided a reference for the development of BPS and its related officinal skincare products.

## 2 Materials and Methods

### 2.1 Materials and Reagents

The fresh fruit of *B. hispida* was purchased from a supermarket in Fengyang County of Anhui province, China. The breed name of *B. hispida* is “Giant No. 2 black-skinned wax gourd”, and its planting process mainly includes several steps including germination, planting with reasonable dense, organic fertilization, erecting vines, removing buds, and harvesting. This product is free of heavy metals including Lead, Cadmium, Mercury, and Arsenic (detected by using Atomic Absorption Spectrophotometry and Atomic Fluorescence Spectrophotometry) and pesticides. Human dermal fibroblasts (HDF-1) were obtained from the National Collection of Authenticated Cell Cultures (Shanghai, China). 1,1-diphenyl-2-picrylhydrazyl (DPPH) and 2,2-azino-bis(3-ethylbenzothiazoline-6-sulphonic acid) (ABTS) were purchased from Aladdin Co. (Shanghai, China). 3-(4,5-dimethyl-2-thiazolyl)-2,5-diphenyl-2-H-tetrazolium bromide (MTT) was obtained from Solarbio Bioscience & Technology Co., Ltd. (Beijing, China). Diethylaminoethyl (DEAE) Sepharose Fast Flow gel was purchased from GE Co. (St. Louis, MO, United States). The fetal bovine serum (FBS) and RPMI 1640 culture medium were provided by Gibco Co. (Carlsbad, CA, United States). Glutathione peroxidase (GSH-Px), superoxide dismutase (SOD), malondialdehyde (MDA), catalase (CAT), transforming growth factor-β1 (TGF-β1), free fatty acid (FFA), tumor necrosis factor (TNF-α), and CCK-8 detection kits were purchased from Nanjing Jiancheng Bioengineering Institute (Nanjing, China). Reactive oxygen species (ROS) detection kit was purchased from Solarbio Technology Co., Ltd. (Beijing, China). Nuclear factor (erythroid-derived 2)-like 2 (NRF2) and heme oxygenase-1 (HO-1) polyclonal antibodies were obtained from Abcam Co. (MA, United States). B-cell lymphoma-2 (Bcl-2) and Bcl2-associated X protein (Bax) polyclonal antibodies were obtained from Santa Cruz Co. (Texas, United States). All other chemicals and solvents were of analytical grade.

### 2.2 Preparation of Polysaccharides

The fruit of *B. hispida* was dried and pulverized into a fine powder, and then the powder (100 g) was extracted twice (1 h/time) with ultrasound-assisted help (200 W) according to the solid-liquid ratio of 1:8. After centrifugation (5,000 r/min, 10 min), the supernatants from the two extractions were combined and rotary-evaporated. Afterwards, the supernatant was precipitated with an equal volume of 75% ethanol overnight at 4°C. After centrifugation (5,000 r/min, 10 min), the precipitate was collected and dissolved in deionized water. Next, 15% trichloroacetic acid was added to remove protein. After centrifugation, the supernatant was collected, dialyzed (cutoff Mw 3,500 Da) for 48 h, and lyophilized to obtain crude polysaccharide samples (8.75 g). The purification of polysaccharides was carried out according to the description by Chen et al. (Chen et al., 2020). Briefly, crude polysaccharide samples (200 mg) were dissolved in deionized water (50 mg/ml) and passed through a 0.45 μm filter. Sample solution was fractionated by DEAE Sepharose Fast Flow gel on a column (2.5 × 60 cm). The sample solution was eluted with a 2 ml/min flow rate and a step gradient (0, 0.10, 0.15, and 0.20 M NaCl). The eluate was collected under monitoring by the phenol-sulfuric acid method. Sample solution was dialyzed against deionized water and lyophilized (37.34 mg).

### 2.3 Chemical Characterization

#### 2.3.1 Preliminary Chemical Composition

The total sugar content was determined by the phenol-sulfuric acid method (Masuko et al., 2005). The uronic acid content was measured by the mhydroxy diphenyl method (Bitter and Muir, 1962). The soluble protein content was estimated by the Bradford method (Bradford, 1976). The polyphenol content was determined by the Folin-Ciocalteu method regarding the national standard method of China (GBT8313-2018).

#### 2.3.2 Molecular Weight (Mw) Distribution

The homogeneity and Mw distribution were carried out according to the procedure used by Li et al. (Li et al., 2015). In short, high-performance size exclusion chromatography (HPSEC) equipped with an eight-angle laser light scattering detector (MALLS, Wyatt Technology Corp, United States) and a refractive index detector (RI). A UV detector was employed to evaluate the weight-average (Mw). The sample concentration was 2 mg/ml, the flow rate was 0.5 ml/min, the injection volume was 100 μl, and the column temperature was kept at 35°C.

#### 2.3.3 Monosaccharide Composition

The monosaccharide composition of the purified PBS was performed regarding the report of Zhu et al. (Zhu et al., 2019; Zhu et al., 2020b) with slight modification. Briefly, 10 mg of polysaccharide sample was dissolved in 4 ml of trifluoroacetic acid (4 M), sealed with nitrogen, and hydrolyzed at 120°C for 4 h. After that, the hydrolyzed solution was concentrated and repeatedly evaporated to dryness by adding methanol. The concentrate was diluted to 100 ml with deionized water, and then the sample was measured by ion chromatography (IC).

#### 2.3.4 Fourier-Transform Infrared (FT-IR) Analysis

A total of 2 mg of PBS sample was thoroughly mixed with 100 mg of KBr powder, compressed into a flaky shape (1-mm thick), and scanned with a PE-1730 infrared spectrometer (Massachusetts, United States) under a wavenumber range of 4,000–400 cm^−1^.

#### 2.3.5 Scanning Electron Microscopy (SEM) Analysis

A total of 2 mg of polysaccharide sample was attached to the metal block with conductive adhesive and a layer of gold was sprayed on the surface of the sample, and then the apparent morphology (500 ×) was analyzed by a HITACHSU8010 scanning electron microscope (JEOL, Tokyo, Japan).

### 2.4 *In vitro* Antioxidant Assay

The *in vitro* antioxidant assays, including DPPH, ABTS, hydroxyl radical (OH¯), superoxide anion (SOA) radical scavenging assays, were conducted based on the procedure reported by Zhu et al. (Zhu, et al., 2021b), and vitamin C (Vc) was applied for the positive control.

### 2.5 *In vitro* Moisture Absorption and Moisture Retention

#### 2.5.1 *In vitro* Moisture Absorption

The BPS sample (1 g) and glycerol (1 g) in the drying dish were respectively dried at 105°C to constant weight. After cooling to room temperature, the samples were transferred to a closed desiccator (25°C, 81% relative humidity) containing saturated ammonium sulfate solution. The mass change of the drying dish and the sample was measured and recorded every 1 h, and the moisture absorption rate was calculated according to Eq. 1.

Moisture absorption rate= [(Ma – M0)/(Mb – M0)] × 100% (1)

Where M0 is the weight (g) of the drying dish with constant mass and the sample, Ma is the weight (g) of the drying dish and the sample after ‘a’ hour, and Mb is the initial weight (g) of the drying dish and the sample before putting them into the desiccator containing saturated ammonium sulfate.

#### 2.5.2 *In vitro* Moisture Retention

The BPS sample and glycerol in the drying dish were respectively dried at 105°C to constant weight. After cooling to room temperature, four groups were designated A, B, C and D as follows: (A) 0.5 g BPS +0.5 g (deionized) water; (B) 0.5 g glycerol +0.5 g water; (C) 0.25 g BPS +0.25 g glycerol +0.5 g water; (D) 0.5 g water. The prepared four groups of samples were transferred to a closed desiccator (25°C, relative humidity below 40%) equipped with discolored silica gel, and the weight change of each group was measured every 1 h, and the moisture retention rate was calculated according to Eq. 2.

Moisture  retention rate= [1 − (0.5 − Ma)/0.5] × 100% (2)

### 2.6 Animal Experiments and Designs

All animal treatments were employed in line with the Experimental Animal Ethics Standards of the Experimental Animal Ethics and Use Committee of Anhui Science and Technology University (approval No. A2020001). Thirty-six female mice were purchased from Qinglongshan Animal Breeding Farm (Nanjing, China), and they were divided into six groups, including Model, glycerol, L-BPS, M-BPS, H-BPS, and Normal groups. After acclimatization for 1 week, the backs of mice were depilated with electric clippers to expose the skin area of 3 × 3 cm^2^. Except for the Normal group, which was injected subcutaneously with saline, the other groups were subcutaneously injected with D-galactose at a dose of 500 mg/kg/d for 2 weeks. The skin aging model was successfully established when the exposed area of the skin after continuous injection became duller, wrinkled, and dry. A total of 10% glycerol solution was applied to the exposed area of the back of the mice in the glycerol group, and BPS solution at doses of 2.5, 5.0, and 10 mg/ml was applied to the exposed area of the back of the mice in the L-BPS, M-BPS and H-BPS groups, respectively. The exposed area on the back of the mice in the model group was not treated. All the mice in the treatment groups were smeared on the back to form a water film on the skin. After treatment for 6 weeks, the mice were euthanized with CO_2_ and their skin was cleaned, and the skin was collected for further analysis. The moisture content of mouse skin was determined by the atmospheric drying method (GB/T 5009.3-2010). A specific volume of normal saline was added to the mouse skin tissue to prepare a 10% homogenate, and then the supernatant was obtained by centrifugation. The MDA level and enzyme activity of SOD and CAT in the supernatant after centrifugation were determined according to the instruction of the commercial kits.

### 2.7 Moisturizing and Antioxidant Evaluation Under Peroxidation Damage

#### 2.7.1 Cell Viability

HDF-1 cells in the exponential growth phase were seeded in a 96-well plate at a density of 1 × 10^4^ cells/well and cultured with RPMI 1640 culture medium containing 15% FBS for 2 h. Subsequently, a series of concentration gradients of BPS samples (50, 100, 250, 500, 750, and 1,000 μg/ml) was added and incubated for 48 h, and the untreated group was regarded as the Control group. After that, cell viability was determined according to the instruction of the CCK8 kit.

#### 2.7.2 Detection of ROS

The H_2_O_2_-induced cellular peroxidation experiment was carried out according to the method of Xu et al. (Chen et al., 2020) with some modifications. Briefly, HDF-1 cells in the exponential growth phase were seeded in a 6-well plate at a density of 1 × 10^5^ cells/well and incubated with different concentration gradients of BPS including L-BPS, M-BPS, and H-BPS groups (100, 250, and 500 μg/ml) for 24 h. Next, 2 ml of 2 μM H_2_O_2_ solution was added and incubated for 1 h. The cells without H_2_O_2_ treatment were regarded as the Normal group, and the cells treated with H_2_O_2_ but no sample was regarded as the Control group. The ROS level in the cells was detected according to the instruction of the ROS detection kits.

#### 2.7.3 Real-Time PCR and Western Blot Assays

The H_2_O_2_-induced peroxidative damage model was constructed as described in chapter 2.7.2. The total RNA was extracted from cells and reverse transcribed into cDNA by using Servicebio®RT First Strand cDNA Synthesis Kit instructions (Service, Wuhan, China). The mRNA expression was assessed by SYBR qPCR Master Mix (High ROX, Wuhan, China) regarding the light quantitative PCR kit instructions, with the following primers:

Primer *AQP3* Forward: CCT​TTG​GCT​TTG​CTG​TCA​CTC
Primer *AQP3* Reverse: ACG​GGG​TTG​TTG​TAA​GGG​TCA
Primer *NRF2* Forward: ACG​GTA​TGC​AAC​AGG​ACA​TTG​AGC
Primer *NRF2* Reverse: TTG​GCT​TCT​GGA​CTT​GGA​ACC​ATG
Primer *NQO1* Forward: AACCAACAGAGCCAATC
Primer *NQO1* Reverse: CCTCCATCCTTTCCTC
Primer *HO-1* Forward: TGC​CAG​TGC​CAC​CAA​GTT​CAA​G
Primer *HO-1* Reverse: TGT​TGA​GCA​GGA​ACG​CAG​TCT​TG
Primer *COL1A1* Forward: GGG​ATT​CCC​TGG​ACC​TAA​AG
Primer *COL1A1* Reverse: GGAACACCTCGCTCTCCA
Primer *ELN* Forward: CAAGGCTGCCAAGTACGG
Primer *ELN* Reverse: CCA​GGA​ACT​AAC​CCA​AAC​TGG
Primer *MMP1* Forward: GGG​AAT​AAG​TAC​TGG​GCT​GTT​CAG
Primer *MMP1* Reverse: CCT​TTG​CAG​ATG​TCT​TTC​CTG​AA

The total protein from cells was extracted and separated, and then electroblotted onto PVDF membranes. After being blocked for 1 h in 5% BSA, it was incubated with the corresponding primary antibodies (1:500-1:1,000 dilution) at 4°C overnight. After incubation with a secondary antibody labeled with horseradish peroxidase for 30 min at room temperature, the conjugates were visualized with an Image analyzer quantitative system (Stepone plus, ABI, California, United States). Quantitative grayscale analysis of protein expression was performed by using AlphaEase FC software (Alpha Innotech, CA, United States).

#### 2.7.4 Biochemical Parameters

The levels of MDA, FFA, TNF-α, and TGF-β1 and the enzymic activities of SOD, CAT, and GSH-Px were determined by using commercially corresponding available kits.

### 2.8 Statistical Analysis

All data are presented as means ± standard deviation with at least triplicate repetitions. GraphPad Prism 8.0 (GraphPad Software Inc., San Diego, CA, United States) was applied for the statistical analysis, which was performed by one-way ANOVA for multiple groups using the Tukey test. A *p*-value less than 0.05 was regarded as significant.

## 3 Results

### 3.1 Preliminary Physicochemical Property of BPS

**FIGURE 1:**
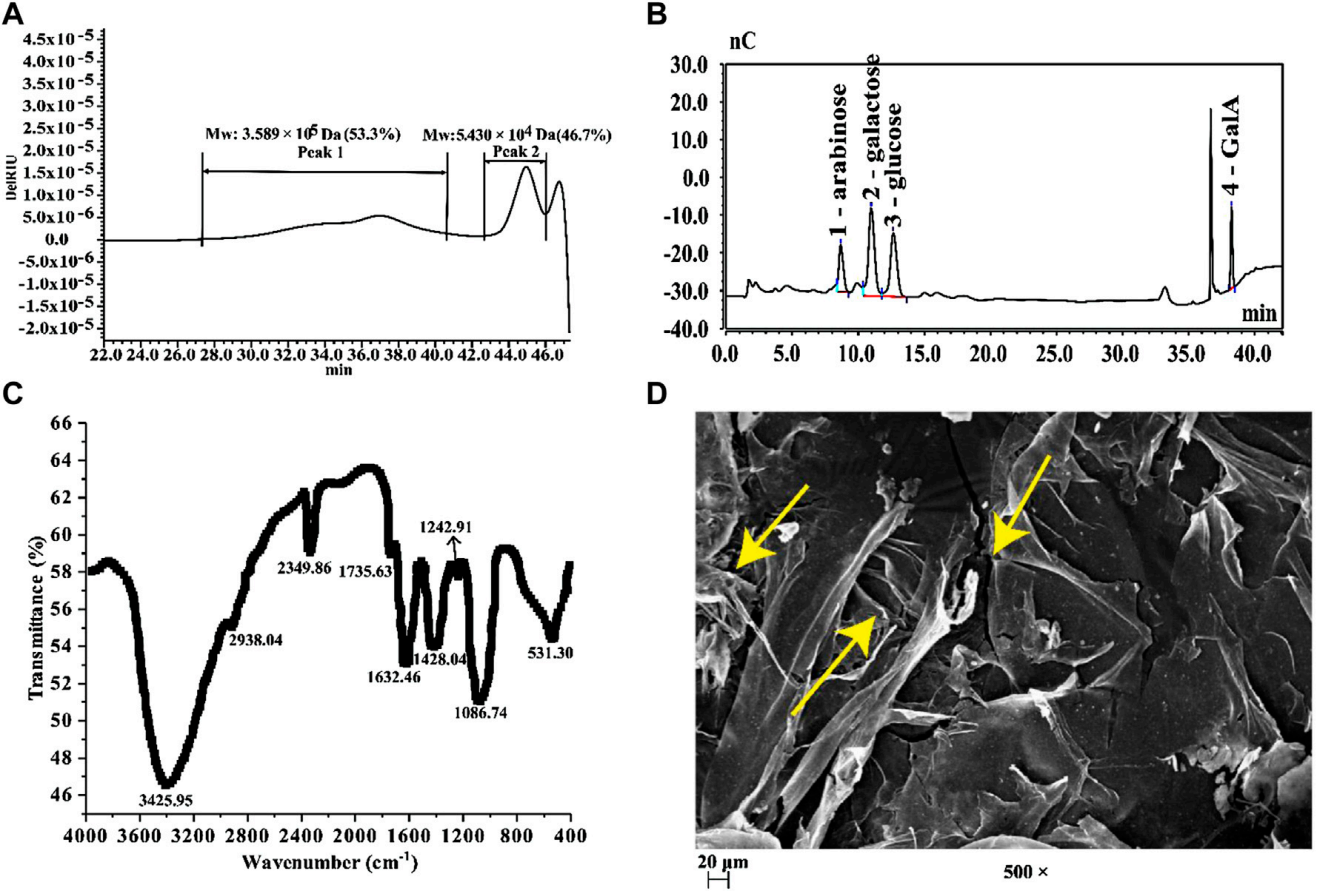
**(A)** HPSEC profiles of BPS; **(B)** IC chromatography of BPS; **(C)** FT-IR spectra of BPS; **(D)** SEM of BPS (× 500).

After ultrasonic-assisted extraction, a fraction was obtained by elution with deionized water using Sepharose FF gel, but other gradients of NaCl elution got no fraction. Also, its yield was 18.67%. The chemical composition of the obtained fraction was analyzed (Table 1). Our results show that the prepared BPS (the BPS mentioned below is a purified sample) contained 94.41% total sugar and 3.66% water-soluble protein, indicating that some binding proteins in BPS may still exist even after the separation and removal of impurities (Zhu et al., 2021b). In addition, 23.42% uronic acid was found in BPS but no polyphenol was detected, which indicates that BPS may be an acidic polysaccharide. The result of Mw distribution (Figure 1A) showed that BPS consisted of two polysaccharides with Mw distributions, namely 3.589 × 10^5^ Da (53.3%) and 5.430 × 10^4^ Da (46.7%). Since the obtained BPS was inhomogeneous, we did not analyze its detailed structure. Further monosaccharide composition analysis (Figure 1B) reveals that BPS was composed of four monosaccharides, including arabinose, galactose, glucose, and GalA, with a molar ratio of 9.19:12.11:10.46:10.19. A series of characteristic absorption peaks (3425.95, 2938.04, 1735.63, 1632.46, 1428.04, and 1086.74 cm^−1^) in the FT-IR analysis (Figure 1C) confirm that BPS was an acidic polysaccharide with a small amount of bound protein (Zhu et al., 2019; Zhu et al., 2020a; Zhu et al., 2021b). In addition, SEM analysis reveals that BPS showed a fragmented and loose morphology presented by yellow arrow (Figure 1D).

**TABLE 1:** Chemical composition of purified BPS.

Total Sugar (%)	Uronic acid (%)	Proteins (%)	Polyphenols (%)	Yield (%)
94.41 ± 1.33	23.42 ± 1.05	3.66 ± 0.10	ND	18.67 ± 0.39

ND: not detected.

### 3.2 Evaluation of *in vitro* Anti-Aging and Moisturizing Activities

**FIGURE 2:**
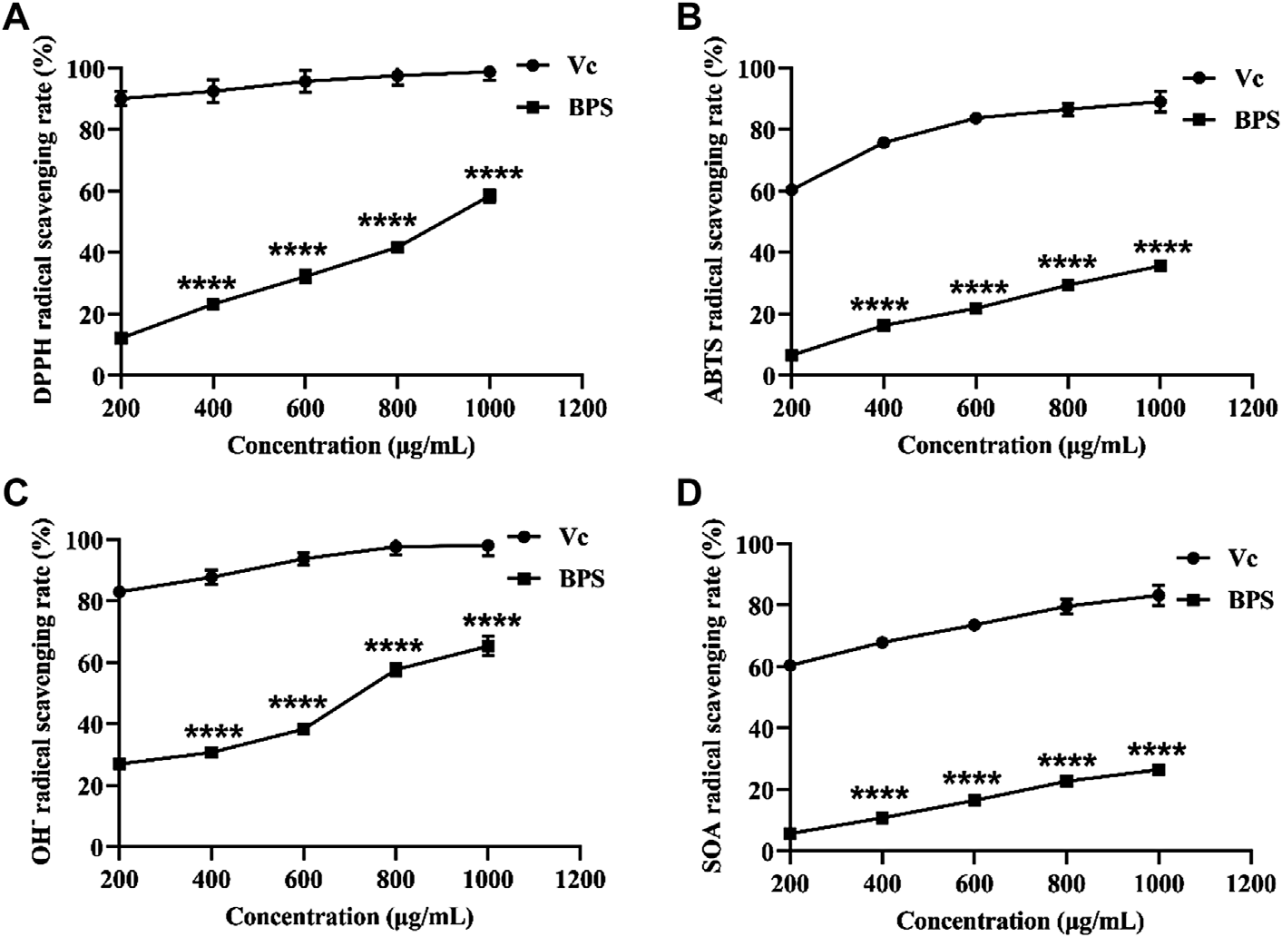
Comparison of *in vitro* anti-aging activities between BPS and Vc, including DPPH radical scavenging activity **(A)**, ABTS radical scavenging activity **(B)**, OH¯ radical scavenging activity **(C)**, and SOA radical scavenging activity **(D)**. The sterisk represents a significant difference compared with the Vc group, ∗∗∗∗*p* < 0.0001. *B. hispida* polysaccharides, BPS; Vc, vitamin C; DPPH, 1,1-diphenyl-2-picrylhydrazyl; ABTS, 2,2-azino-bis(3-ethylbenzothiazoline-6-sulphonic acid); OH¯, hydroxyl radical; SOA, superoxide anion.

Free radicals are the typical products of oxidative metabolism in the human body. Still, excessive accumulation of free radicals will destroy the function of biofilms and even lead to cell lysis and apoptosis (de Francisco et al., 2018). Therefore, eliminating free radicals can reduce their damage to skin cells and delay skin aging. Our results show that BPS scavenged four typical free radicals in a dose-dependent manner (Figures 2A–D). However, its scavenging capability still had a particular gap compared with Vc, even at high concentrations.

**FIGURE 3:**
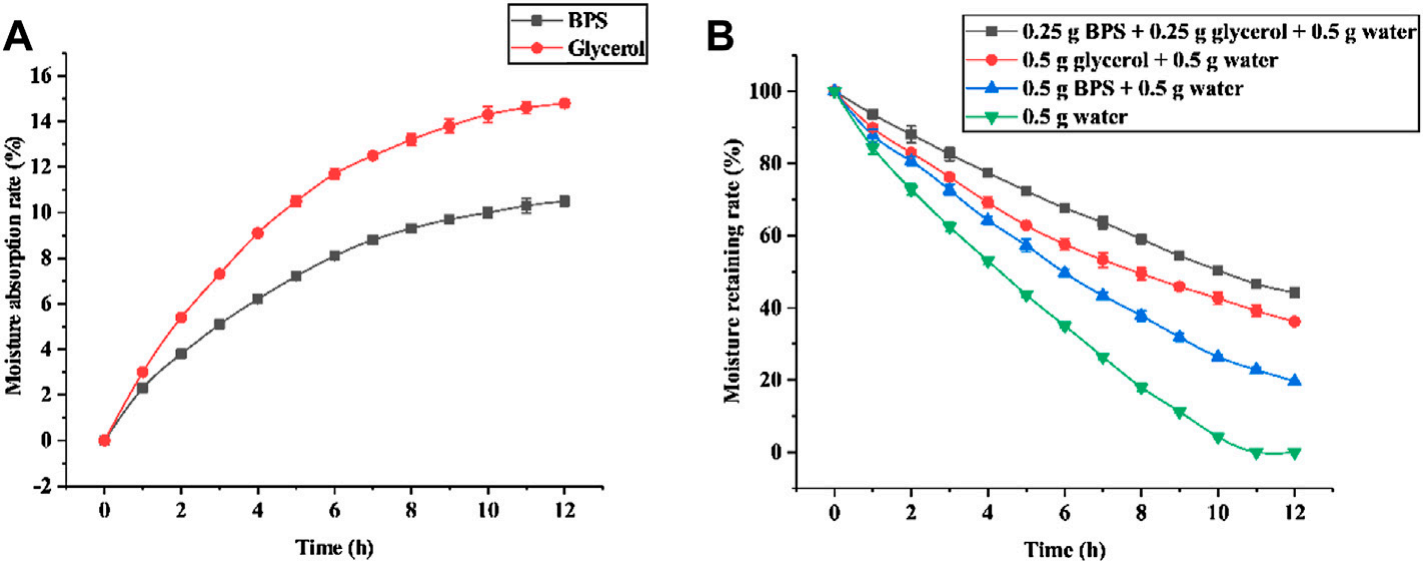
*In vitro* moisture absorption **(A)** and moisture retention **(B)** of BPS.

With the increase of time (0–12 h), the moisture absorption rate of BPS after drying was gradually increased, and the growth rate of moisture absorption rate was first fast and then slow (Figure 3A). At 12 h, the moisture absorption rate of BPS reached the maximum value (10.5%). Glycerol, which is recognized as an excellent hygroscopic agent, has a better hygroscopic effect than BPS within 0–12 h. However, the moisture absorption rate of BPS at 12 h was 70.95% of that of glycerin, indicating that BPS has prominent *in vitro* hygroscopic capability. In addition to moisture absorption, moisture retention is also an indispensable effect of skincare products. Under the condition of 25°C and relative humidity of 40%, the moisture retention rate of 0.50 g water decreased most obviously, followed by 0.50 g BPS +0.50 g water, 0.50 g glycerol +0.50 g water, whereas 0.25 g BPS +0.25 g glycerol +0.50 g water had an excellent long-term moisturizing effect. The moisture retention rate for 12 h was 44.20% (Figure 3B).

### 3.3 Animal Model Evaluation of Anti-Aging and Moisturizing Activities

**FIGURE 4:**
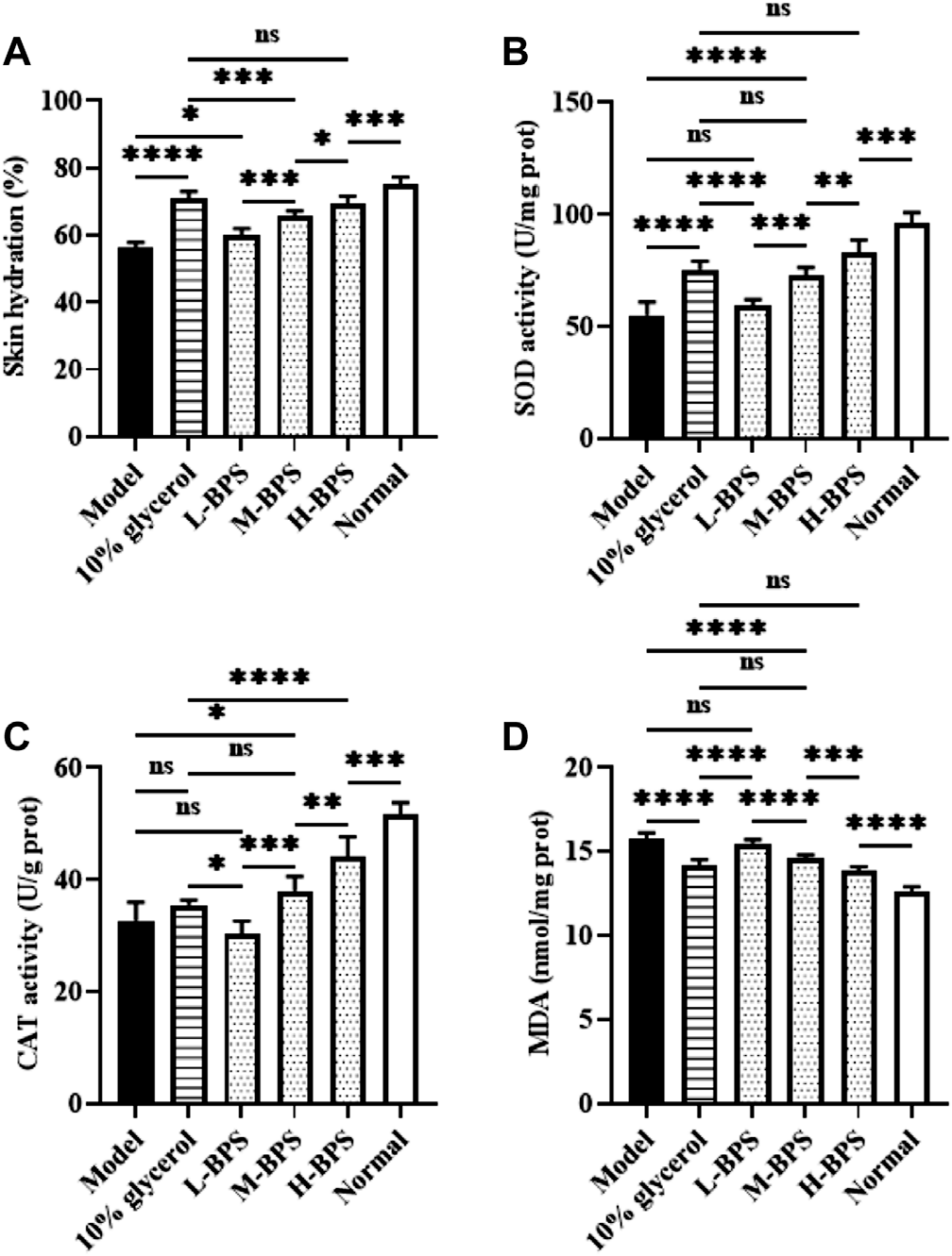
Effects of BPS treatment on skin hydration **(A)**, SOD activity **(B)**, CAT activity **(C)**, and MDA level **(D)** of mouse skin in a skin aging mouse model. ∗, *p* < 0.05; ∗∗, *p* < 0.01; ∗∗∗, *p* < 0.001; ∗∗∗∗, *p* < 0.0001; ns, not significant.

With the progression of age, skin aging aggravates the reduction of water content in the skin and the accumulation of many free radicals, resulting in weakened differentiation and reduced vitality of skin cells (Csekes and Račková, 2021). Therefore, in addition to *in vitro* moisturizing and anti-aging activities, we also investigated *in vivo* moisturizing and anti-aging activities. Figure 4A below illustrates that the skin hydration after treatment with BPS was significantly increased with BPS concentration. Additionally, SOD and CAT activities were also significantly elevated and the MDA level was greatly decreased (Figures 4B–D).

### 3.4 Cellular Model Evaluation of the Anti-Aging and Moisturizing Mechanism

#### 3.4.1 Intracellular ROS Scavenging

**FIGURE 5:**
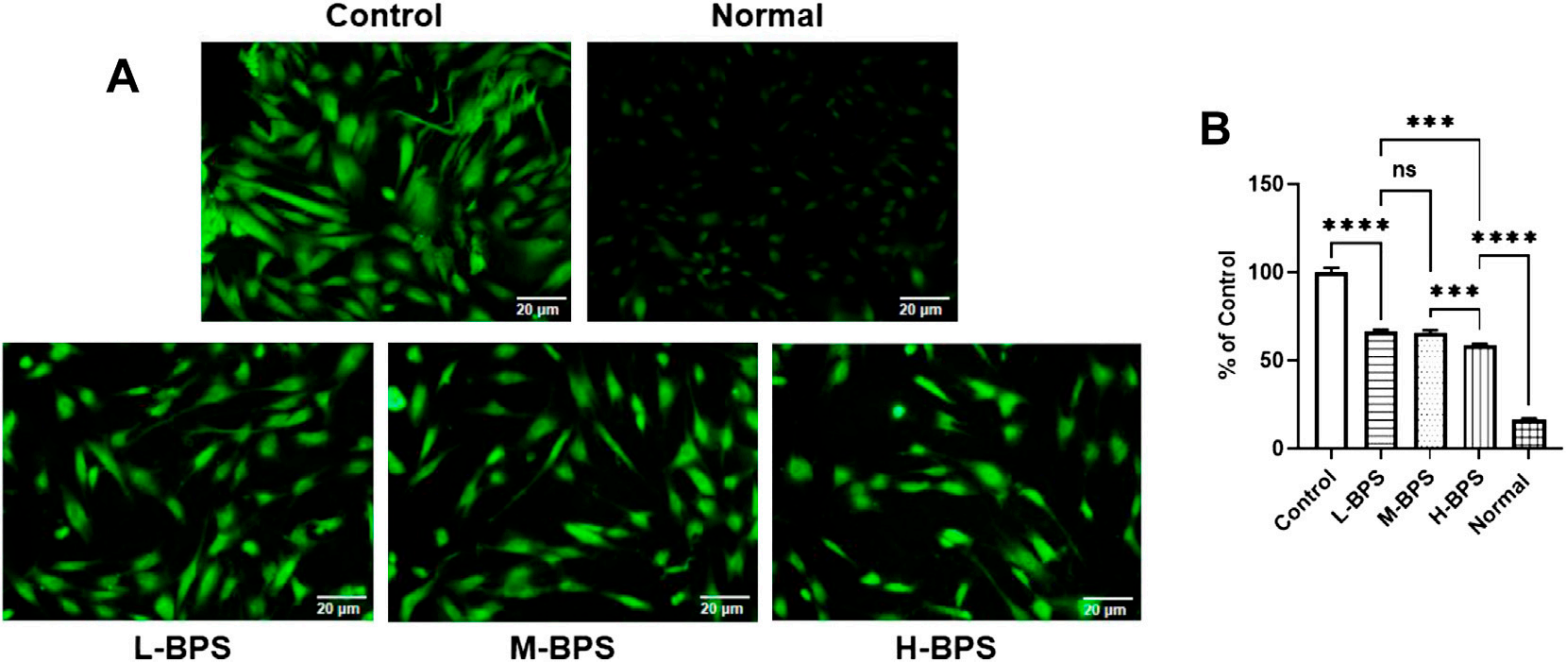
ROS distribution in HDF-1 cells under fluorescence microscope **(A)**. Quantitative analysis of intracellular ROS concentration (compared to the Control group) by using a multifunctional fluorescence microplate reader at excitation and emission wavelengths of 488 and 533 nm **(B)**. L-BPS, M-BPS, and H-BPS represent a BPS-treated concentration of 100, 250, 500 μg/ml, respectively. Asterisk indicates a significant difference between two groups. ∗∗∗, *p* < 0.001; ∗∗∗∗, *p* < 0.0001; ns, not significant.

**FIGURE 6:**
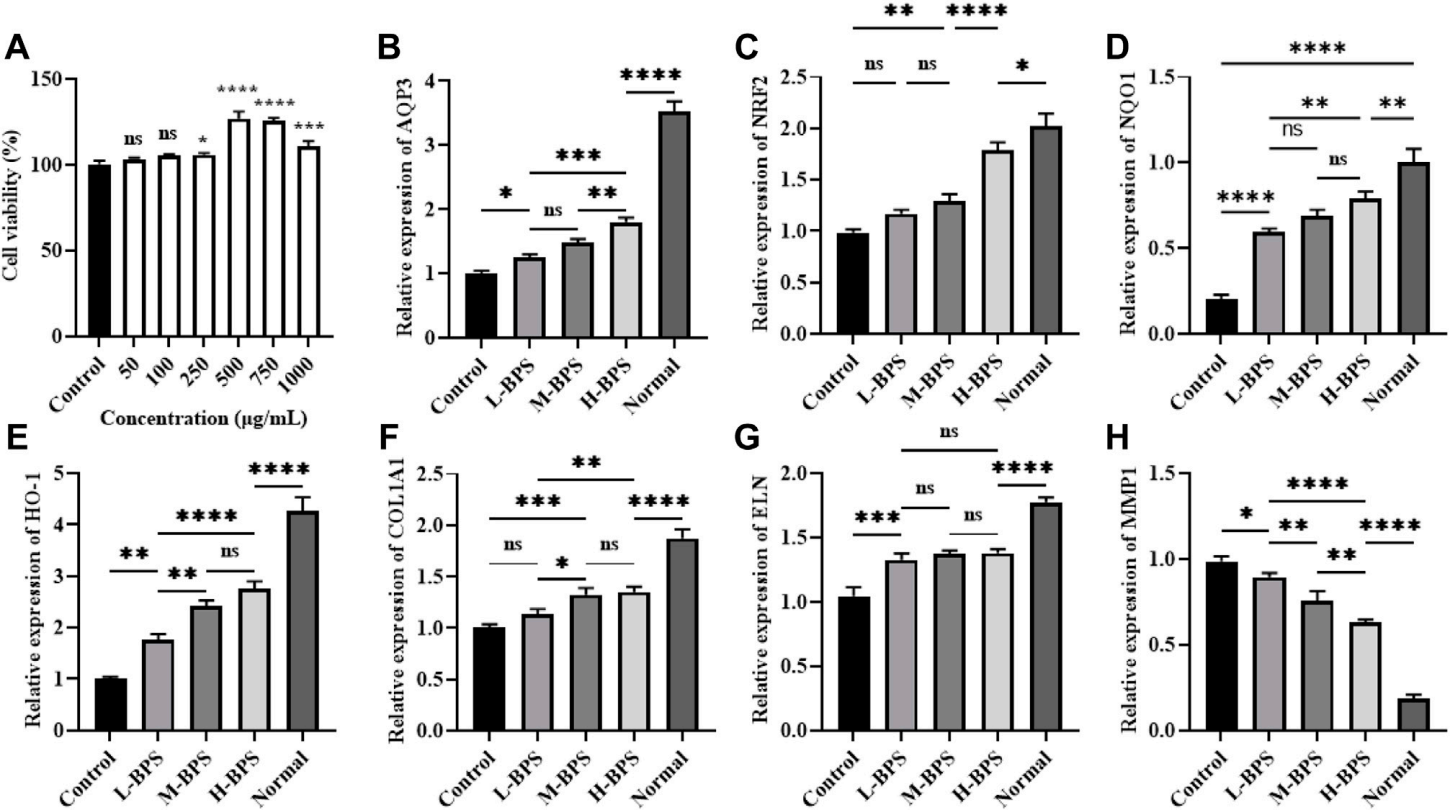
Effects of different concentrations of BPS on cell viability **(A)** and related moisturizing and anti-aging gene expression levels including *AQP3* **(B)**, *NRF2* **(C)**, *NQO1* **(D)**, *HO-1*, **(E)** *COL1A1* **(F)**, *ELN* **(G)**, and *MMP1* **(H)** in H_2_O_2_-treated HDF-1 cells. ∗, *p* < 0.05; ∗∗, *p* < 0.01; ∗∗∗, *p* < 0.001; ∗∗∗∗, *p* < 0.0001; ns, not significant.

To further analyze the possible mechanisms of moisturizing and anti-aging in skincare activities, we constructed a cellular model of H_2_O_2_-induced free radical damage. As shown in Figures 5, 6A, cytotoxicity was not found by treatment with BPS and H_2_O_2_ treatment significantly increased the ROS level in HDF-1 cells. However, BPS supplementation prevented the increased level of ROS and showed a dose-dependent relationship.

#### 3.4.2 Regulation of Related Anti-Aging and Moisturizing Gene Expression Levels by BPS

To analyze the possible mechanism by which BPS regulates intracellular ROS levels, we analyzed the effect of BPS on the expression levels of moisturizing and antioxidant-related genes. BPS increased the H_2_O_2_-induced decrease of *AQP3* (Figure 6B) mRNA level in a concentration-dependent manner. Also, *NRF2*, *HO-1*, and *NQO1* mRNA levels were significantly increased by incubation with BPS (Figures 6C–E). Moreover, *COL1A1* and *ELN* mRNA levels were also elevated and the MMP1 mRNA level was reduced by BPS regulation (Figures 6F–H).

#### 3.4.3 Regulation of Related Anti-Aging Protein Expression Level by BPS

**FIGURE 7:**
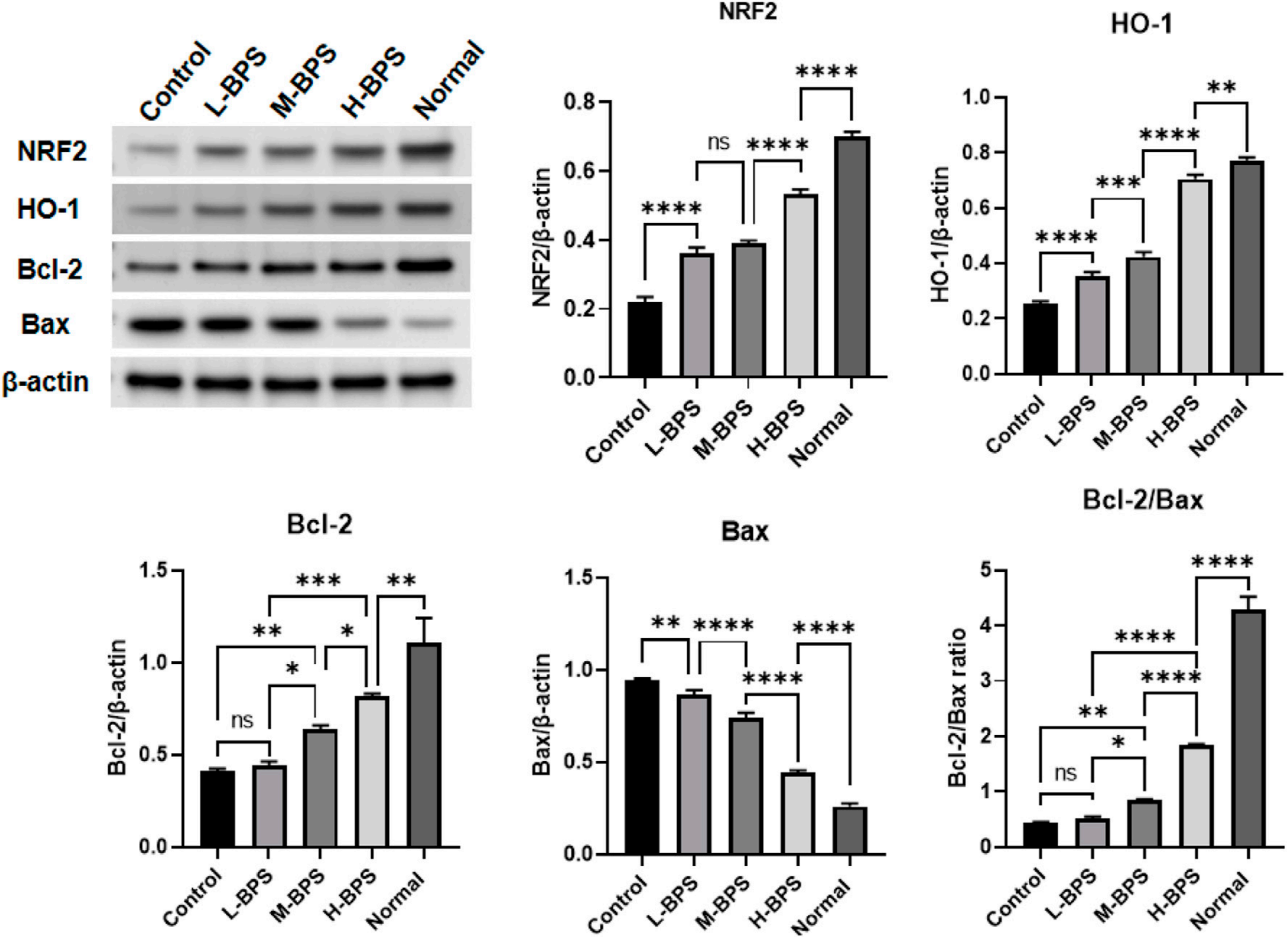
Effects of BPS on related anti-aging protein expression levels including NRF2, HO-1, Bcl-2, and Bax in H_2_O_2_-treated HDF-1 cells. ∗, *p* < 0.05; ∗∗, *p* < 0.01; ∗∗∗, *p* < 0.001; ∗∗∗∗, *p* < 0.0001; ns, not significant.

As mentioned above, we also investigated the effects of BPS on the expression levels of antioxidant-related proteins. NRF2, HO-1, and Bcl-2/Bax protein levels were decreased by H_2_O_2_-induced treatment and prevented by simultaneous incubation with different concentrations of BPS (Figure 7).

#### 3.4.4 Regulation of Related Anti-Aging Related Cytokine Levels and Related Enzyme Activities

**FIGURE 8:**
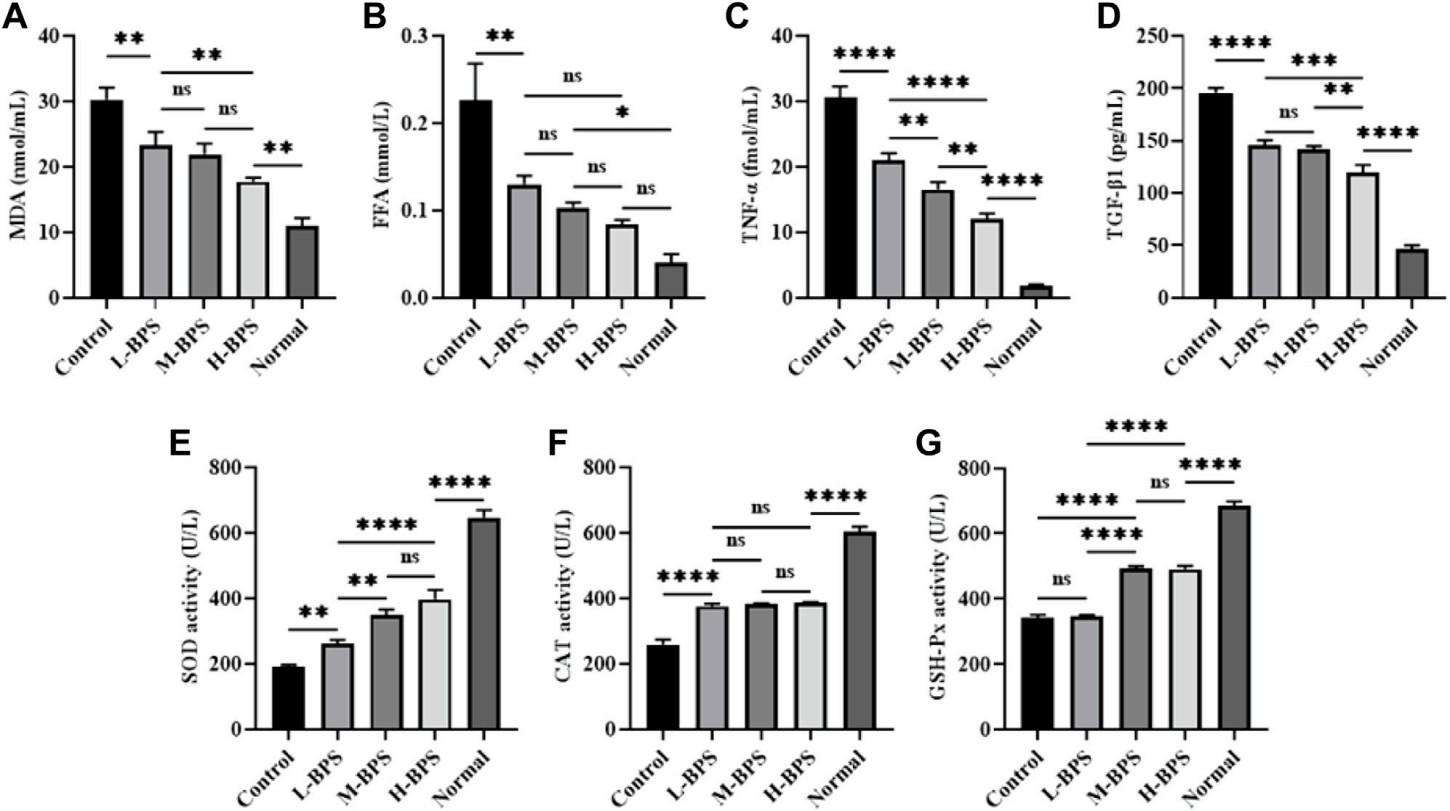
Effects of BPS on related cytokine levels including MDA **(A)**, FFA **(B)**, TNF-α**(C)**, and TGF-β1 **(D)** and related enzyme activities including SOD **(E)**, CAT **(F)**, and GSH-Px **(G)** in H_2_O_2_-treated HDF-1 cells. ∗, *p* < 0.05; ∗∗, *p* < 0.01; ∗∗∗, *p* < 0.001; ∗∗∗∗, *p* < 0.0001; ns, not significant.

In addition to related anti-aging genes and proteins, we also measured related cytokine levels and critical enzyme activities, and the results are presented in Figure 8. As can be seen, the incubation of H_2_O_2_ led to higher levels of MDA, FFA, TNF-α, and TGF-β1, and this increase was suppressed by simultaneous incubation with different concentrations of BPS (Figures 8A–D). Moreover, the decrease in SOD, CAT, and GSH-Px activities evoked by H_2_O_2_ was reversed by incubation with different concentrations of BPS (Figures 8E–G).

## 4 Discussion

As the body’s first barrier organ, the skin is susceptible to various stimuli that affect its form and function. Senescent cells have been found to accumulate with age and may contribute to progressive age-related skin aging (Csekes and Račková, 2021). The aging of the global population not only presents an increasing demand for strategies to prevent and treat skin-related diseases but also for skincare products containing natural ingredients capable of alleviating skin aging. Due to the few side effects and favorable efficacy of natural products, functional skincare ingredients derived from nature have recently received extensive attention. Natural polysaccharides have many health benefits, including the skin’s anti-aging (Du et al., 2014). By affecting the molecular pathways of aging, polysaccharides can prevent or delay the development of aging, thereby avoiding or improving skin aging and age-related lesions (Du et al., 2014). In this study, we found that natural BPS exhibited favorable moisturizing and anti-aging effects, related to its unique physicochemical properties. Moreover, BPS could significantly activate the NRF2/HO-1 signaling pathway, thereby alleviating H_2_O_2_-evoked peroxidative damage in fibroblasts.

The fruit of *B. hispida* contains massive water-soluble polysaccharides, and it has shown strong free radical scavenging capability, but the specific reason is not clear (Jiang et al., 2016). It may be a feasible attempt to analyze its antioxidant capacity from physicochemical properties. The considerable content of galactose and GalA in BPS may highlight the strong bioactivity of BPS (Solomon et al., 2015; Zhang et al., 2015; Zhu et al., 2021b). In addition, the fragmented and loose morphology of BPS (Figure 1D) laid a specific foundation for its favorable bioactivity (Zhu et al., 2021b).

Aging is one of the risk factors for many skin-related diseases, and this characteristic can be explained by the most popular theory of free radicals (Pomatto and Davies, 2018). However, unbalanced body metabolism makes the free radicals produced unable to be effectively scavenged. As a result, the accumulation of a large amount of extracellular free radicals in the skin’s dermis accelerates the peroxidation of the skin, resulting in damage to cell differentiation and metabolic functions and ultimately rapid aging of human skin (Hekimi et al., 2011). Our results show that BPS exhibited specific free radical scavenging capabilities in a concentration-dependent manner, especially against DPPH and OH- radicals. Consequently, we speculate that BPS was more inclined to preferentially donate hydrogen rather than electrons to exhibit its radical-scavenging effect (Chen et al., 2020). On the one hand, low-Mw BPS is less viscous and easier to contact and scavenge free radicals (Zhu et al., 2020a). Meanwhile, low-Mw BPS has more reducing ends, which can better react with free radicals and exert a quenching effect (Zhu et al., 2021b). On the other hand, BPS with higher uronic acid content is more likely to activate anomeric hydrogen atoms to react with free radicals (Wu et al., 2013). The findings of the current study are consistent with those of Chen and Yang who found that plant polysaccharides had some degree of free radical scavenging capability (Yang et al., 2017; Chen et al., 2020), which provides the possibility for the development of BPS-related anti-aging products.

The stratum corneum (SC) is the outermost layer of the skin, protecting the body from external factors and controlling exchanges with the environment, especially transepithelial water loss (Celleno, 2018). Hygroscopic molecules enable SC to retain water, thereby keeping the *epidermis* moist and elastic (Celleno, 2018). Therefore, skin humidity is the most essential condition to ensure skin rejuvenation. Multiple studies have shown that maintaining skin moisture is essential to protect skin barrier function and provide the flexibility of the stratum corneum (Cha et al., 2021). As a favorable moisturizer, polysaccharide efficacy has been reported in several studies (Kanlayavattanakul et al., 2017; Cha et al., 2021). Our outcomes reveal that BPS presented a potent *in vitro* moisturizing effect. The main factors affecting the hygroscopicity and moisturizing properties of macromolecular compounds are the number of hydrophilic groups in their structure and the strength of their hydrophilicity. Therefore, we speculate that a large number of hydroxyl groups in BPS enhanced its hygroscopicity, whereas the polar groups (such as hydroxyl and carboxyl) in BPS form strong hydrogen bonds with water, which enhances *in vitro* moisture retention (Li et al., 2017). As well as *in vitro* moisturizing, the continued BPS treatment significantly improved skin hydration in galactose-induced aging mice compared to the Model group. Further results indicate that BPS also boosted the *AQP3* expression level in H_2_O_2_-induced HDF-1 cells. The most important cells in the skin’s dermis are HDF cells, and the *AQP3* gene in them is closely related to skin moisturizing function and elasticity, responsible for transporting water and uncharged molecules on the cell membrane (Schrader et al., 2012). Studies have shown that *AQP3* can bring water, glycerol, and triglycerides from the sebaceous glands into the *epidermis*, preventing epidermal drying and promoting epidermal hydration (Schrader et al., 2012). Our study implies that BPS might exert *in vivo* moisturizing activity by enhancing the expression of the *AQP3* gene in the skin.

HDF cells’ number, morphology, secretion, and synthesis function are closely related to skin aging. Free radicals mainly cause the aging of the body, and the stimulation of H_2_O_2_ may lead to the apoptosis of HDFs (Cai et al., 2016). The decomposition of H_2_O_2_ will cause the degradation of macromolecules such as intracellular hyaluronic acid and protein and continuously generate OH- free radicals, which will lead to the production of new H_2_O_2_ and trigger a chain reaction of oxidative stimulation (Valachová et al., 2016). Under the stimulation of H_2_O_2_, the excessive production of ROS destroys the cellular defense barrier and triggers oxidative stress, which causes irreversible damage to mitochondria and causes damage to cell structure and function (Sies and Jones, 2020). ROS mainly comes from oxidative cell metabolism and plays a major role in chronological ageing and skin photoaging (Noh et al., 2016). Further metabolic changes may be indicated by the findings of a cellular senescence model established using HDF cell lines. Our results show that BPS could exert protective and antioxidant effects on H_2_O_2_-induced damage to HDF-1 cells by reducing the generation of intracellular ROS. Alternatively, there are similarities expressed by Xu et al. and those described by Han et al. (Han et al., 2017; Xu et al., 2021). We consider that the data presented here will comprise a critical discussion point for future studies of polysaccharides against ROS.

NRF2 is a vital transcription factor sensitive to oxidants and plays a crucial role in regulating cellular oxidative responses through oxidative stress defense mechanisms (Sakurai et al., 2018). Its activation can regulate the transcription of many genes with cytoprotective and detoxifying effects, such as HO-1 and NQO1, to maintain cellular redox homeostasis and attenuate cellular oxidative stress (Quincozes-Santos et al., 2013). HO-1 can catalyze the rate-limiting step of heme catabolism, resulting in the formation of bilirubin, free iron, and carbon monoxide, thereby enhancing cell resistance to oxidative damage (He et al., 2014). NQO-1 protects cell membranes from oxidative damage by reducing endogenous quinine compounds (Li et al., 2019). After exposure to ROS, NRF2 migrates into the nucleus, binds to the antioxidant response element region, and initiates transcription of target genes. Fibroblasts isolated from NRF2-knockout mice were reported to exhibit increased levels of intracellular ROS and increased sensitivity to oxidative stress (Loboda et al., 2016). In addition, studies show that pretreatment with resveratrol could attenuate oxidative damage by up-regulating the expressions of NRF2, HO-1, and NQO1 (Shen et al., 2016). This study showed that the mRNA and protein expression levels of *NRF2* and *HO-1* were significantly decreased in HDF-1 cells after H_2_O_2_-induced ROS production to form peroxidative damage. In addition, the mRNA expression levels of *NQO1*, *COL1A1*, and *KLN* were also significantly increased and the mRNA expression level of *MMP1* was noticeably decreased. *COL1A1* is a key gene that controls the production of type I collagen, whereas matrix metalloproteinases have a degrading effect on collagen, and *MMP1* is the main related gene (Park et al., 2018). Besides, *ELN* is a key gene that controls skin elastin production (Park et al., 2018). The above three genes are closely associated with skin aging. These findings suggest that the NRF/HO-1 signaling pathway might be important after peroxidative damage of aging. The NRF2/HO-1 signaling pathway can be activated by most phytochemical extracts or their derivatives, including polysaccharides (Zhao et al., 2019; Zhang et al., 2021). Therefore, our findings suggest that the NRF2/HO-1 signaling pathway was crucial in the peroxidative protection of BPS against H_2_O_2_ injury.

An important component of the apoptotic pathway is the Bcl-2 family of proteins. The main role of Bcl-2 family members is to regulate apoptosis, and Bax is a protein that promotes apoptosis (Siddiqui et al., 2015). The Bcl-2/Bax ratio has been reported as an essential determinant of apoptosis (Siddiqui et al., 2015). BPS treatment not only activated the NRF2/HO-1 pathway but also significantly up-regulated the Bcl-2/Bax ratio, so we propose that BPS plays a protective effect on HDF-1 under oxidative stress by activating the NRF2/HO-1 pathway. In addition, the significantly reduced ROS content in the BPS-treated group also showed the protective effect of enhanced NRF2/HO-1 expression against oxidative stress injury. Therefore, the results of this part confirmed that improving the expression of the NRF/HO-1 pathway could mediate the anti-apoptotic effect of senescent cells caused by peroxidation.

The ROS production during the senescence will cause DNA damage. Both SOD and CAT are important antioxidant enzymes, which can effectively remove free radicals generated in cell metabolism (Csekes and Račková, 2021). MDA is the degradation product of lipid peroxides formed by oxidation reactions in the body, which can cause metabolic dysfunction and even apoptosis of cells (Wang et al., 2017). MDA is a product of lipid peroxidation and an important marker of oxidative stress. Also, all cells have defense functions to resist peroxidative damage and protect cells from ROS damage, these members include SOD, CAT, GSH-Px (Shen et al., 2016). SOD can catalyze the disproportionation of superoxide anion into oxygen and hydrogen peroxide, CAT can decompose hydrogen peroxide into water and oxygen, and GSH-Px can convert toxic peroxide into non-toxic peroxide by redox glutathione (Zhu et al., 2021a). In this study, the activities of related antioxidant enzymes including SOD, CAT, and GSH-Px in H_2_O_2_-induced HDF-1 cells or galactose-evoked aging mice were significantly decreased, and the MDA level was greatly increased. Compared with the Control group, BPS treatment significantly enhanced the activities of SOD, CAT, and GSH-Px and noticeably decreased the MDA level. Consistent with our report, a study reported by Liao et al. indicates that the polysaccharide from Okra could improve the antioxidant capacity of type 2 diabetic mice by enhancing the activities of SOD, CAT, and GSH-Px (Liao et al., 2019). Similarly, Lin et al. found that the polysaccharide from *Cyclocarya paliurus* could enhance the antioxidant defense system by up-regulating the activities of SOD, CAT, and GSH-Px (Lin et al., 2020). The aging of the global population not only presents an increasing demand for strategies to prevent and treat age-related diseases but also for skincare products containing natural ingredients with active ingredients capable of combating skin aging. So far, there are few related skincare products developed by BPS. This study indicates that BPS may exhibit a strong skincare activity in the skin aging model and have a great potential to develop into natural skincare products.

Senescent cells induced by excess ROS are characterized by their inability to proliferate, resist apoptosis, and secrete factors that promote inflammation and tissue deterioration (Höhn et al., 2017), which alter the local tissue environment or lead to chronic inflammation. In addition to regulating oxidative stress factors, BPS also significantly down-regulated the levels of inflammatory factors including FFA, TNF-α, and TGF-β1. Elevated levels of inflammatory cytokines are not limited to age-related systemic changes but may also be associated with skin aging. Notably, senescent cells that accumulate in the skin during aging play a significant role in driving skin inflammation (Pilkington et al., 2021). The anti-inflammatory properties of naturally active polysaccharides have been widely recognized (Muszyńska et al., 2018), and our results have demonstrated this again. Currently, there is still a lack of universal biomarkers of aging. Our results suggest that FFA, TNF-α, and TGF-β1 may be potential candidates as markers of aging.

## 5 Conclusion

Our results suggest that BPS had favorable moisturizing and anti-aging properties. Also, BPS pretreatment could attenuate H_2_O_2_-induced peroxidative damage, which might be related to the activation of the NRF2/HO-1 pathway. In the future, more studies are needed to explore the specific mechanisms by which NRF2/HO-1 pathway regulates various antioxidant enzymes and moisturizing functions.

## Data Availability

The original contributions presented in the study are included in the article/Supplementary Material, further inquiries can be directed to the corresponding authors.
